# Explicit Associative Learning and Memory in Synesthetes and Nonsynesthetes

**DOI:** 10.1177/2041669516658488

**Published:** 2016-09-15

**Authors:** Kaitlyn R. Bankieris, Richard N. Aslin

**Affiliations:** University of Rochester, USA

**Keywords:** long-term memory, memory, multisensory or cross-modal processing, synaesthesia

## Abstract

Most current theories regarding the development of synesthesia focus on cross-modal neural connections and genetic underpinnings, but recent evidence has revitalized the potential role of associative learning. In the present study, we compared synesthetes’ and controls’ ability to explicitly learn shape-color pairings. Using a continuous measure of accuracy and multiple testing blocks, we found that synesthetes learned these pairings faster than controls. In a delayed retest, synesthetes outperformed controls, demonstrating enhanced long-term memory for shape–color associations. Following this retest, participants learned shuffled associations, and we found little evidence for group differences in subsequent learning ability. Overall, our findings support the hypothesis that synesthetes have exceptional associative learning abilities and further specify that this advantage pertains to the initial learning rate and long-term retention of associations.

Synesthesia is a phenomenon in which perceptual or cognitive stimuli (e.g., the word *January*) automatically and consistently elicit additional, unusual percepts (e.g., the color chartreuse). Although early hypotheses regarding the causal mechanism for synesthesia focused on associative learning (Calkins, 1893; Claparede, 1903), this explanation has since lost interest (see Yon & Press, 2014 for a discussion). Current hypotheses favor cross-modal neural connections (e.g., Grossenbacher & Lovelace, 2001; Ramachandran & Hubbard, 2001) and genetic underpinnings (e.g., Asher et al., 2009) as causes for synesthesia, but recent evidence has revitalized the potential role of *associative* learning in the development of synesthesia (for a comprehensive review of the role of learning in synesthesia, see Watson, Akins, Spiker, Crawford, & Enns, 2014).

Supporting the role of associative learning, Witthoft and Winawer (2013) reported evidence that early experience contributes to the associations of 11 grapheme-color synesthetes. The grapheme-color pairings of these synesthetes largely overlapped with the colors of toy letters and numbers to which they were exposed as children. Moreover, this research group recently conducted a large-scale study investigating the influence of such toys on 6,588 grapheme-color synesthetes’ associations (Witthoft, Winawer, & Eagleman, 2015). Results demonstrated that toys with colored graphemes influenced a large number of synesthetic associations (10–26 per individual) for approximately 6% of synesthetes. In the years preceding the introduction of a specific colored toy set to the commercial market, no participants’ synesthetic experiences had a large overlap with the colored toys. In the decade following the availability of this toy set to consumers, however, nearly 15% of participants exhibited significant overlap between their synesthetic experiences and the toy set. These results suggest a causal link between exposure to a particular set of environmental stimuli and the formation of synesthetic associations, suggesting that associative learning in early childhood plays a role in synesthesia.

Additional findings support the role of environmental statistics in synesthesia. Although synesthetic experiences are superficially idiosyncratic from one synesthete to the next (e.g., the letter *a* might be red for one synesthete but green for another), many types of synesthesia reflect patterns found in the general population (see Simner, 2013 for review). Sound-color synesthetes, for example, tend to associate higher pitches with lighter colors, and nonsynesthetes tend to favor this same mapping when asked to make intuitive judgments in forced-choice cross-sensory association tasks (Marks, 1974; Ward, Huckstep, & Tsakanikos, 2006). Many forms of synesthesia follow this same general principle of reflecting nonsynesthetes’ implicit associations (e.g., Bankieris & Simner, 2014; Cytowic & Wood, 1982; Marks, 1974, 1987; Simner & Ludwig, 2012; Simner et al., 2005; Smilek, Carriere, Dixon, & Merikle, 2007). These common patterns across synesthetes and nonsynesthetes suggest that a sensitivity to associative pairings or environmental statistics—particularly early in development—may play a part in the formation of synesthetic associations.

Despite the foregoing evidence, a handful of studies investigating synesthetes’ ability to explicitly learn associations have been conducted and provide mixed evidence for superior associative learning. For instance, Brang, Ghiam, and Ramachandran (2013) required grapheme-color synesthetes and controls to learn novel grapheme-color pairings and found that learning rate did not differ between groups and that synesthetes demonstrated worse maintenance of the learned pairings. In a similar experiment, Rothen and Meier (2010) trained 44 grapheme-color synesthetes to associate simple line drawings with colors using the Weschler Memory Scale—Revised Visual Paired Associates Test. Synesthetes exhibited enhanced memory both when immediately retested and after a delay of at least 30 minutes. Pritchard, Rothen, Coolbear, and Ward (2013) added a layer of difficulty to this standard shape–color pairing task by requiring participants to additionally remember the location of items in a 5 × 2 grid. They found that grapheme-color synesthetes were better than controls at this task by the end of the study but exhibited the same learning rate as controls. Finally, Pfeifer, Rothen, Ward, Chan, and Sigala (2014) tested grapheme-color synesthetes and controls on a fractal–fractal association task and found no group differences for time to reach criterion or accuracy. Overall, these studies provide mixed evidence that synesthetes have an enhanced ability to explicitly learn associations, but it is possible that these experimental designs were not optimal to address this question. For example, the forced-choice design and binary accuracy measures used in these studies may not be sensitive enough to detect learning differences between synesthetes and controls. Additionally, no existing studies have looked at both rate of learning and long-term maintenance within the same task. Finally, allowing participants to direct their own exposure to and learning of the associations may reveal differences in learning strategies that lead to enhanced learning for synesthetes.

The current study addresses the possibility that synesthetes have an increased sensitivity to environmentally present associations with a design that targets specific known facets of synesthesia. First, synesthesia is not explicitly taught. Our design does explicitly ask participants to learn shape–color pairings but allows them to control their exposure to each pairing in an attempt to create a slightly more naturalistic experience. Second, synesthesia is defined in part by the consistency of associations over time. Accordingly, we elicit specific color choices (Hue-Saturation-Brightness values) as the continuous dependent measure instead of using a limited palette and recording accuracy as a binary measure. Not only do we frequently test remembered associations within an experimental session to ascertain a smooth learning curve, but we also repeat testing across days and weeks to investigate potential long-term memory differences between synesthetes and controls. A necessary side effect of having consistent associations over time is that synesthetes do not generally learn new associations throughout life. To test whether synesthetes’ differ from controls in their ability to learn new associations, we shuffled the shape–color pairings (same shapes, same colors, and different pairings) during their final session. With this multi-part experimental design, we sought to investigate synesthetes’ ability to explicitly learn, retain, and relearn shape–color associations.

## Methods

### Participants

A total of 14 synesthetes (mean age = 26.7, *SD* = 11.6, 4 males) experiencing colors for letters, numbers, days of the week, and months of the year were recruited from our existing database of Rochester area synesthetes. A total of 15 nonsynesthetes (mean age = 20.1, *SD* = 3.0, 7 males) were recruited from the Rochester area. All participants were compensated $10/hour for their participation. Ethical approval was obtained from the University of Rochester Research Subjects Review Board.

All recruited synesthetes’ self-reported experiences were previously confirmed with an objective test of genuineness—consistency over time—presented via the diagnostic website synesthete.org (see Eagleman, Kagan, Nelson, Sagaram, & Sarma, 2007 for methods). This test identifies synesthetes based on replicated findings that synesthetes are significantly more consistent when repeatedly choosing synesthetic colors for the stimuli eliciting them (e.g., letters) compared with nonsynesthetes. Our synesthetes experienced colors in response to graphemes (*n* = 10), days of the week (*n* = 10), and months of the year (*n* = 8) as confirmed by mean standardized scores of .51 (*SD* = .20), .54 (*SD* = .18), and .53 (*SD* = .16), respectively, where a score below one confirms synesthesia (see Eagleman et al., 2007 for details). Five synesthetes experienced colors for graphemes, days of the week, and months of the year; three had synesthetic colors for only days of the week and months of the year; four were solely grapheme-color synesthetes; two synesthetes experienced colors for only days of the week. Nonsynesthetes completed a synesthesia questionnaire (see synesthete.org) on paper, indicated no synesthetic experiences, and were further verbally questioned to ensure a complete lack of such experiences.

### Stimuli

Stimuli consisted of nine discriminable “snowflake” shapes created with ART-TEK’s online snowflake generator (http://www.art-tek-ltd.co.uk/snowflake/). We selected the nine most perceptually discriminable colors (in terms of Euclidean CIE distance) from each other and our gray background as the target colors.

### Procedure

**Figure 1.**
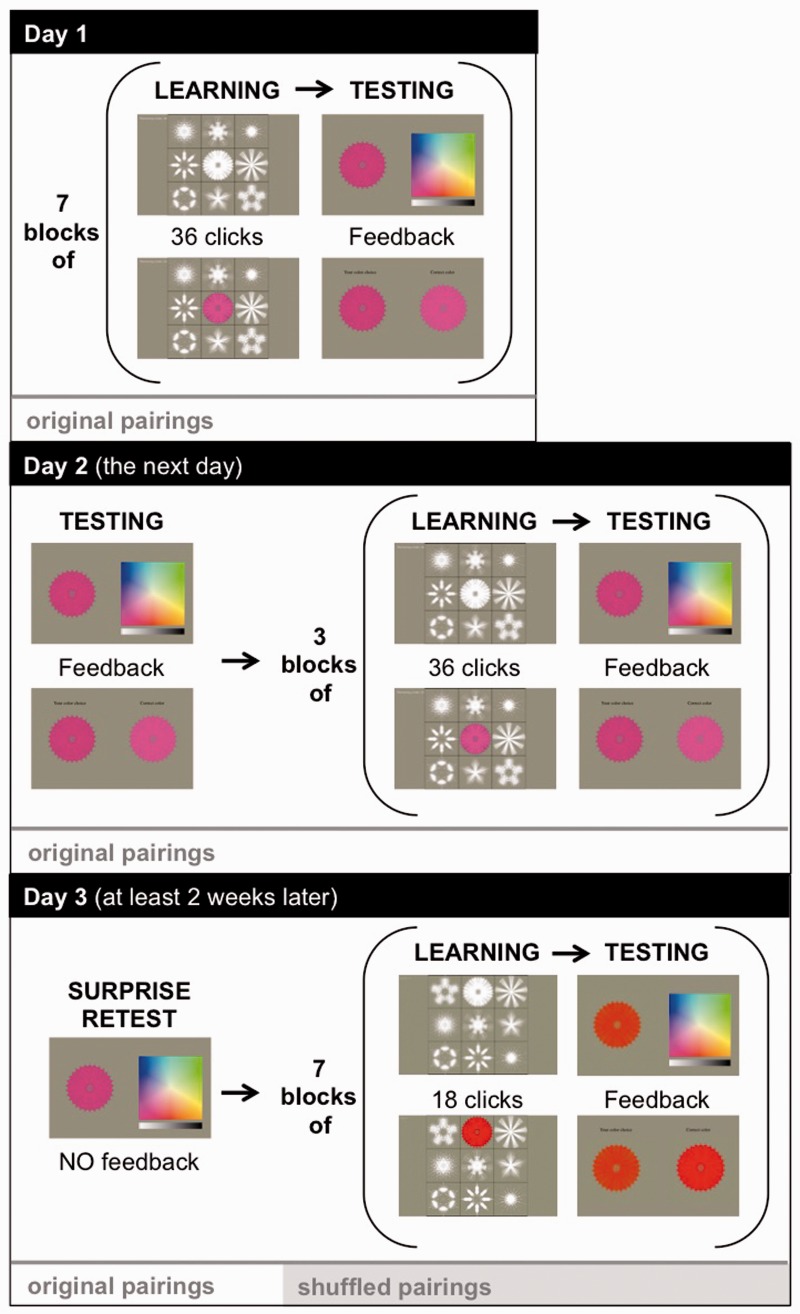
Experimental procedure.

Participants learned nine snowflake–color pairings across three in-lab sessions as shown in Figure 1. At the beginning of Session 1, participants completed a pretest in which they were shown all nine white snowflakes sequentially and asked if they automatically and consistently associated a color with that snowflake. We excluded snowflakes for which participants had existing color associations from our analyses. This pretest further confirmed the lack of synesthetic experiences in our controls since none of the controls reported a preexisting color association with a snowflake. After the pretest, participants completed seven blocks of learning and testing interleaved. Learning phases presented all nine snowflakes in white, randomly positioned in a 3 × 3 grid on a touch screen. The position of snowflakes in this grid was randomized for each learning block to remove snowflake location as a potential cue. Participants touched any snowflake to see the target color that was randomly assigned to it. The selected snowflake then turned its assigned color for 2 seconds. Each learning block consisted of 36 snowflake touches distributed, however, the participant chose. Testing phases presented each snowflake one at a time in white alongside a color picker and a luminance slider. Participants touched the color square to paint the snowflake its learned color, submitted their answer when satisfied, and were immediately shown the snowflake in their chosen color next to the snowflake in its assigned color as feedback. The first and last testing phases of visits one and two presented each snowflake twice; all other testing phases presented each snowflake once.

The second visit to the lab occurred the next day and began with a testing phase. The remainder of the second visit consisted of three training and testing phases interleaved and a computerized version (Pebl) of the Corsi-block tapping task to assess working memory. At least 2 weeks after the initial visit, we contacted the participants and asked them to come back into the lab for a third and final visit. They were not told that they would be retested on the associations that they learned previously. This visit began with a testing phase requiring participants to recall the snowflake–color associations learned previously (each snowflake was presented four times without feedback). Then, the snowflake–color pairings were shuffled and participants learned the new pairings across seven learning and testing phases interleaved (with learning phases reduced to 18 touches).

## Results

We conducted a mixture model analysis on each feature of participant color responses (hue, saturation, and brightness) to tease apart the two types of memory required for this task: knowing that a particular snowflake shape should be paired with a particular color (e.g., red) and accurately remembering the precise shade of that color.

We modeled error for each color feature (hue, saturation, and brightness) with respect to a probabilistic model of memory performance initially described by Zhang and Luck (2008) and expanded by Bays, Catalao, and Husain (2009) and Bays, Wu, and Husain (2011). This model as applied to our experimental design proposes that color responses come from three potential sources: (a) correctly reproducing the color of the *target* item with variability (target component T), (b) mistakenly reproducing the color of a *nontarget* item with variability (nontarget component N), and (c) randomly guessing a color (random component R). Accordingly, a general mixture model (McLachlan & Peel, 2000) with three components describes the distribution of responses: $p(\overset{\wedge }{\theta })=\Sigma_{k}\alpha_{k}p_{k}$ where $\overset{\wedge }{\theta }$ is the color response, $\alpha_{k}$is the probability that a response comes from the *k*th component, and *p_k_* is the probability density function describing the response distribution under that component. The probability density functions of our three components are shown in Table 1, with variability for hue errors following von Mises distributions (because hue is a circularly distributed variable), while the response variability for saturation and brightness errors follow Gaussian distributions (because these are not circularly distributed variables). We used full Bayesian statistical inference via Markov Chain Monte Carlo to obtain estimates of the mixture parameters $\left\{\alpha_{T},\alpha_{N},\alpha_{R}\right\}$, as well as σ, the standard deviation of the von Mises or Gaussian distribution, for each block (1–19) and group (synesthete, control) using the rStan package (Stan Development Team, 2015). We ran separate models for each color feature (hue, saturation, and brightness).

**Table 1.** Mixture Model Components Describing the Response Distribution for a Single Color Feature.

	Probability density $p_{k}$	Response type
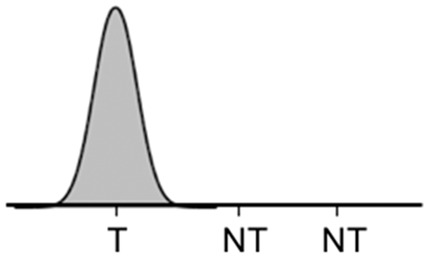	$N_{\sigma }(\mu -y)$	Target
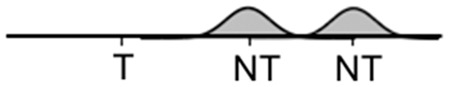	$\frac{1}{d}\sum_{i}^{d}N_{\sigma }(\mu_{i}-y)$	Non-target
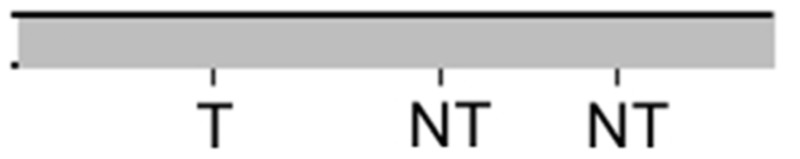	$\frac{1}{510}$	Random

*Note.* μ represents the true color value of the target snowflake, and *y* is the feature value reported by the participant (range 1–255 for saturation and brightness, −π to π for hue). μ*i* is the true feature value of the *i*th nontarget snowflake (*d* in total). $N_{\sigma }$ is the Gaussian distribution (von Mises for hue) with *M* = 0 and *SD* = σ. The uniform component is described by 1/2π for hue responses. In the diagrams depicting response probability, the true feature values for the target snowflake (T) and nontarget snowflakes (NT) are indicated (for illustration purposes, only two nontargets are shown).

For each of these three analyses, we ask the following four questions (looking for the effects in parentheses):

1. Do synesthetes learn original pairings more quickly than nonsynesthetes? (group effect for first block of original pairings)
2. Do synesthetes retain original pairings with greater accuracy after a long-term delay? (group effect for the retest)
3. Do synesthetes have greater interference when learning shuffled pairings? (group effect on slope between retest and first block of shuffled pairings)
4. Do synesthetes learn shuffled pairings more quickly than nonsynesthetes? (group effect for first block of shuffled pairings)

Analyses included only snowflakes for which participants did not report a preexisting synesthetic association (233/261).

### Learning Original Pairings

**Figure 2.**
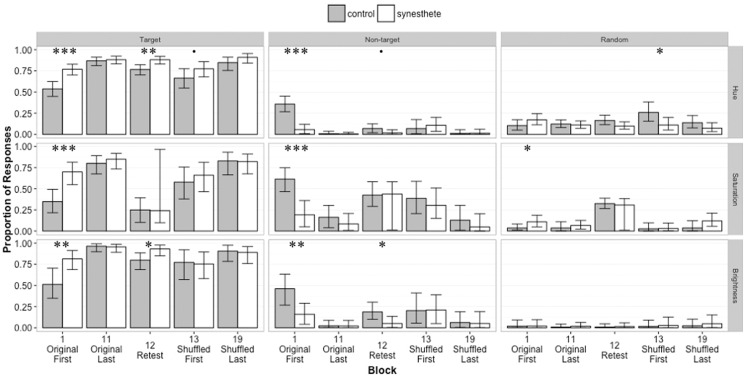
Distribution of responses for each color feature and group. Medians and 95% credible intervals are plotted. Data from all 19 blocks can be found in supplementary material. Asterisks indicate statistical significance of group differences: *** *p* < .001, ** *p* < .01, * *p* < .05, • *p* < .1.

To determine whether group membership affected the distribution of participant responses across components during the initial learning of associations, we compared the groups’ three mixture model parameters $\alpha_{T},\alpha_{N},\alpha_{R}$ for Block 1 obtained by the hue, saturation, and brightness models (see Figure 2). We report the medians of parameter estimates along with MCMC “*p* values” as the percentage of parameter estimate posterior samples that do not follow the trend reported (i.e., *p* = .05 for a trend of *x*>*y* denotes that 5% *x*<*y*). Results from the hue mixture model revealed significant group differences for target and nontarget responses. Synesthetes’ response distribution for hue consisted of more “target” responses ($\alpha_{\mathrm{TS}}$ = 0.77, $\alpha_{\mathrm{TC}}$ = 0.54, *p* < .001) and fewer “nontarget” responses than controls ($\alpha_{\mathrm{NS}}$ = 0.06, $\alpha_{\mathrm{NC}}$ = 0.36, *p* < .001). We found no difference in the proportion of “random” hue responses across groups; $\alpha_{\mathrm{RS}}$ = 0.10, $\alpha_{\mathrm{RC}}$ = 0.17, *ns*. Analyzing saturation responses, we found a similar pattern of results: synesthetes produced a greater proportion of “target” responses ($\alpha_{\mathrm{TS}}$ = 0.70, $\alpha_{\mathrm{TC}}$ = 0.35, *p* < .001), a smaller proportion of ‘nontarget’ responses ($\alpha_{\mathrm{NS}}$ = 0.19, $\alpha_{\mathrm{NC}}$ = 0.61, *p* < .001), and a greater proportion of “random” responses than controls, $\alpha_{\mathrm{RS}}$ = 0.11, $\alpha_{\mathrm{RC}}$ = 0.04, *p* < .05. Our analysis of brightness responses revealed the same trend found for hue responses. Synesthetes produced a higher proportion of “target” responses ($\alpha_{\mathrm{TS}}$ = 0.81, $\alpha_{\mathrm{TC}}$ = 0.51; *p* < .01) and a lower proportion of “nontarget” responses compared with controls; $\alpha_{\mathrm{NS}}$ = 0.16, $\alpha_{\mathrm{NC}}$ = 0.46, *p* < .01. Groups did not differ in the proportion of “random” brightness responses they produced; $\alpha_{\mathrm{RS}}$= $\alpha_{\mathrm{RC}}$= 0.02, *ns*. These results from the first block of learning of the original snowflake–color associations demonstrate that synesthetes learn these associations faster than nonsynesthetes along all three color features. To ensure that these results demonstrate a learning rate advantage rather than a general advantage for synesthetes, we compared group response distributions on the final block of original associations (Block 11). We found that synesthetes and nonsynesthetes did not have different response distributions for any of the color features at the end of learning the original associations. This pattern of results suggests that synesthetes learn the shape–color associations faster than nonsynesthetes, but not more accurately overall.

These results cannot be accounted for by heightened perceptual abilities among grapheme-color synesthetes for their color judgments (e.g., Yaro & Ward, 2009) because our analysis additionally fits a parameter that models the variance of errors surrounding “target” and “nontarget” values for each group. Indeed, fitting this variance by block and group reveals that synesthetes’ fidelity in reproducing colors is better than that of controls in Block 1 of the experiment for each feature of color (hue: *σ_s_* = 0.21, *σ_c_* = 0.15, *p* < .01; saturation: *σ_s_* = 18.02, *σ_c_* = 23.52, *p* < .001; brightness: *σ_s_* = 58.16, *σ_c_* = 74.82, *p* < .01), but this difference is not significant at the end of learning the original associations (Block 11); hue: *σ_s_* = 0.14, *σ_c_* = 0.14, *ns*; saturation: *σ_s_* = 16.56, *σ_c_* = 17.66, *ns*; brightness: *σ_s_* = 44.52, *σ_c_* = 49.83, *ns*. This pattern of results suggests that synesthetes enter the experiment with a heightened ability to report colors from memory with a color picker but controls catch up when given experience with this task. Synesthetes’ initial fidelity advantage is most likely due to their completion of a nearly identical task when documenting their synesthetic experiences (required to select them for our study).

Since participants self-directed their sampling of the pairings during learning, it is possible that group differences in sampling strategy contributed to group differences in learning rate. To address this possibility, we conducted a mixed-effects linear regression on the learning phase data for original pairings (Blocks 1–7 and 9–11). This model predicted number of snowflake samples (i.e., clicks) during training from the fixed effects of stimulus color, block, group, and all interactions, along with the full random-effects structure by participant. If sampling behavior during the learning phase differed between groups, this would appear as a significant interaction between group and stimulus color or a significant three-way interaction among group, stimulus color, and block. This analysis revealed a significant main effect of stimulus color (*B* = 0.15, *p* < .05) and an interaction of stimulus color and block (*B* = 0.02, *p* < .01), indicating that snowflakes associated with some colors were sampled more than others and that this bias in sampling increased as learning of the original pairings progressed. However, none of the interactions with group were significant, suggesting that synesthetes and controls adopted similar sampling strategies for learning the original pairings.

### Memory for Learned Pairs

To assess long-term retention of the original pairings by group, we examined the parameter estimates for the retest after a 2-week delay. For hue responses within the retest, the mixture model revealed significant group differences for “target” and “random” response types. Synesthetes’ gave a higher proportion of “target” responses ($\alpha_{\mathrm{TS}}$ = 0.88, $\alpha_{\mathrm{TC}}$ = 0.77, *p* < .01) and a lower proportion of “random” responses than controls; $\alpha_{\mathrm{RS}}$ = 0.10, $\alpha_{\mathrm{RC}}$ = 0.16, *p < *.05. Synesthetes also produced marginally fewer “nontarget” responses; $\alpha_{\mathrm{NS}}$ = 0.02, $\alpha_{\mathrm{NC}}$ = 0.07, *p* = .06. We found no group differences for the distribution of saturation responses during the retest; $\alpha_{\mathrm{TS}}$ = 0.24, $\alpha_{\mathrm{TC}}$ = 0.25, *ns*; $\alpha_{\mathrm{NS}}$ = 0.44, $\alpha_{\mathrm{NC}}$ = 0.43, *ns;* $\alpha_{\mathrm{RS}}$ = 0.31, $\alpha_{\mathrm{RC}}$ = 0.32, *ns*. Our analysis of brightness responses revealed group differences for proportion of “target” and “nontarget” responses. Synesthetes produced a higher proportion of “target” responses ($\alpha_{\mathrm{TS}}$ = 0.93, $\alpha_{\mathrm{TC}}$ = 0.80; *p* < .05) and a lower proportion of “nontarget” responses compared with controls ($\alpha_{\mathrm{NS}}$ = 0.05; $\alpha_{\mathrm{NC}}$ = 0.19, *p < *.05. Groups did not differ in the proportion of “random” brightness responses they produced; $\alpha_{\mathrm{RS}}$ = $\alpha_{\mathrm{RC}}$ = 0.01, *ns*. These results from the delayed retest of the original snowflake–color associations demonstrate that synesthetes retain associations across a 2-week delay better than nonsynesthetes.

### Interference for Learning New Pairings

To investigate the interference experienced by participants when learning new snowflake–color pairings, we compared the distribution of responses from the retest of original associations (Block 12) and the first block of new associations (Block 13) for each color component. We computed difference measures for each group and response type by subtracting the parameter estimates for Block 12 from those of Block 13. Then, we compared these difference scores across groups to determine whether the amount of interference differed by group. Our analyses revealed no group differences in the amount of interference experienced when learning new associations. Thus, our results do not support the hypothesis that synesthetes’ have greater interference when learning new associations.

### Learning Rate for New Pairings

Our last analyses of interest compared group responses during the first block of learning the shuffled snowflake-color pairings (Block 13) for hue, saturation, and brightness. For hue responses, we found a marginally significant group difference in proportion of “target” responses, with synesthetes producing more “target” responses than controls; Medians: $\alpha_{\mathrm{TS}}$ = 0.78, $\alpha_{\mathrm{TC}}$ = 0.66; *p* = .07. Our analyses also revealed that controls produced more “random” hue responses than synesthetes; Medians: $\alpha_{\mathrm{RS}}$ = 0.11, $\alpha_{\mathrm{RC}}$ = 0.26, *p* < .05. No group differences were found for the distribution of saturation or brightness responses for the learning of shuffled pairings. These results for the learning of shuffled snowflake–color pairings suggest that synesthetes may learn the new shape–hue parings faster than nonsynesthetes. Again, to ensure that these group differences were not due to the ability to learn the pairings in general, we compared group response distributions for the final block of learning the shuffled associations (Block 19). We found no significant group differences in response distribution for the final block of learning the shuffled associations, suggesting that synesthetes and nonsynesthetes learn these associations equally well, but synesthetes may learn them faster.

### Generalized Group Differences

Finally, we compared group performance on the Corsi block-tapping task to ensure that our synesthetes did not have superior memory in general or increased motivation. A *t* test revealed no significant block span differences between controls and synesthetes; *M_C_* = 6.87, *M_S_* = 6.75, *t*(25) = −0.021, *ns*.

## Discussion

The present study investigated the hypothesis that synesthetes have enhanced associative learning abilities compared with nonsynesthetes using an explicit shape–color learning task. In an attempt to gain a more realistic and fine-grained understanding of synesthetes’ ability to explicitly learn these pairings, we allowed participants to self-direct their exposure to the snowflake–color pairings and collected a continuous accuracy measure at multiple time-points during learning. Additionally, we conducted analyses to tease apart memory for specific colors and memory for newly learned associations between shapes and colors.

Our study in general supports the hypothesis that synesthetes have a superior ability to learn and retain shape–color associations. We found that synesthetes learned initial snowflake–color pairings more quickly than controls, producing more “target” and fewer “nontarget” responses than nonsynesthetes across hue, saturation, and brightness color components. Moreover, we determined that this increased learning rate can neither be attributed to differences in sampling strategy of the snowflake–color pairings nor heightened color perception abilities of synesthetes. A delayed retest of these shape–color associations also revealed that synesthetes’ long-term memory was stronger than nonsynesethetes'. Finally, we found minimal evidence that participants’ ability to learn shuffled snowflake–color pairings differed by group, although we cannot draw strong conclusions from this portion of our findings given the small number of participants who were able to return for this phase of the experiment (synesthetes = 7, controls = 4).

Our results extend previous findings suggesting that synesthetes are better color matchers than nonsynesthetes. We did find that synesthetes’ ability to produce specific colors across hue, saturation, and brightness components was better than that of nonsynesthetes for the first block of the experiment. This finding supports previous studies (e.g., Yaro & Ward, 2009) reporting that synesthetes are better than controls at selecting a color to match a target color swatch. However, we did not find a group difference in color-matching variance when looking at performance later in the experiment (Block 11). This pattern of findings demonstrates that when nonsynesthetes are given ample experience with a color matching task, their ability to accurately reproduce particular colors improves to the level of synesthetes. Given that all of our synesthetes previously completed the synesthesia battery during which they reported their synesthetic colors from memory with nearly the exact same color selection tool, it seems likely that synesthetes’ decreased color variability in Block 1 is a practice effect rather than a superior perceptual ability.

Overall, the present study provides evidence that synesthetes have a heightened ability to explicitly learn and retain shape–color associations. Thus, these findings are pertinent to the synesthesia, learning, and memory literatures. Future research should test synesthetes with associations beyond their synesthetic domain (e.g., not using shapes and colors for grapheme-color synesthetes) to evaluate whether or not their heightened performance is indicative of a general, explicit associative learning ability.

## Supplementary Material

Supplementary material

## Supplementary Material

The online supplementary material is available at http://ipe.sagepub.com/supplemental
