# Supplementary material for: Explicit Associative Learning and Memory in Synesthetes and Nonsynesthetes
Source: Iperception. 2016 Sep 15;7(5):2041669516658488. doi: 10.1177/2041669516658488 (PMC5030759; doi:10.1177/2041669516658488)
Supplement: Supplementary material [file Supplementary_Material.pdf]

Supplementary Material

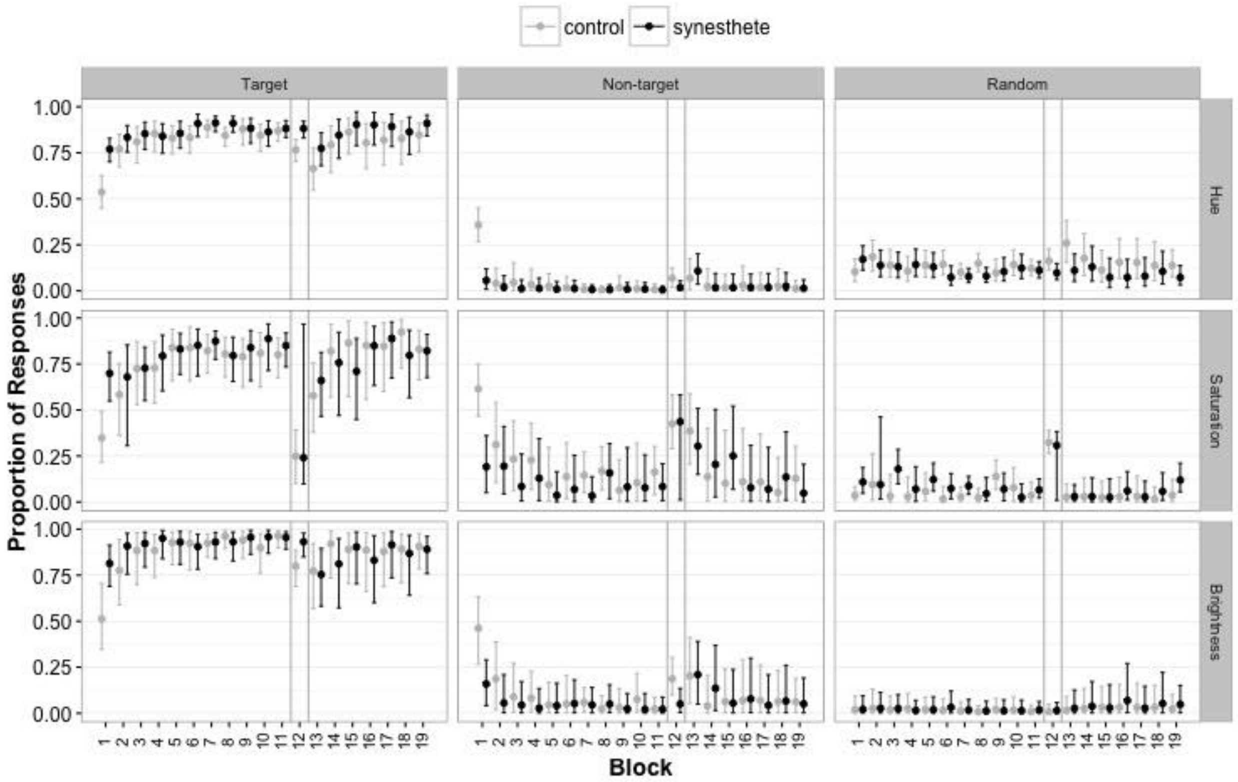

Figure 1S: *Distribution of responses for each color feature and group.* Medians and 95% credible intervals are plotted. Vertical lines separate the learning of the original snowflake-color pairings (blocks 1-11) from the retest (block 12) and the learning of the shuffled snowflake-color pairings (blocks 13-19).
